# Sport Activity and Clinical Outcomes after Hip Arthroscopy with Acetabular Microfractures at a Minimum 2-Year Follow-Up: A Matched-Pair Controlled Study

**DOI:** 10.3390/life12081107

**Published:** 2022-07-22

**Authors:** Mattia Loppini, Francesco La Camera, Francesco Manlio Gambaro, Riccardo Ruggeri, Guido Grappiolo, Federico Della Rocca

**Affiliations:** 1 Department of Biomedical Sciences, Humanitas University, Pieve Emanuele, 20090 Milan, Italy; francescomanlio.gambaro@alumni.hunimed.eu; 2 IRCCS Humanitas Research Hospital, Rozzano, 20089 Milan, Italy; francesco.lacamera@humanitas.it (F.L.C.); riccardo.ruggeri@humanitas.it (R.R.); guido.grappiolo@humanitas.it (G.G.); federico.della_rocca@humanitas.it (F.D.R.); 3 Fondazione Livio Sciutto Onlus, Campus Savona, Università degli Studi di Genova, 17100 Savona, Italy

**Keywords:** hip arthroscopy, chondral lesions, microfractures, return to sport

## Abstract

*Background*: Acetabular microfractures for cartilage lesions have been shown to be a safe procedure able to improve patient reported outcomes (PROs). Nevertheless, the return to sport activity rate represents a crucial outcome to be investigated in these young athletic patients. *Methods*: Patients undergoing acetabular microfracture for full chondral acetabular lesions were compared to a 1:1 matched-pair by age and gender control group undergoing hip arthroscopy without microfractures. Clinical assessment was performed with PROs and participation in sports in terms of type and level of activities was evaluated preoperatively and at 2-years follow-up. *Results*: A total of 62 patients with an average age of 35.1 ± 8.1 (microfracture group) and 36.4 ± 6.3 (control group) were included. In both groups, the average values of PROs significantly increased from preoperatively to the last follow-up. There was no significant difference between the two groups in the number of patients playing at the amateur and elite level preoperatively and at the last follow-up. *Conclusions*: Microfractures for the management of full-thickness acetabular chondral defect provides good clinical results at a minimum follow-up of two years, which are not inferior to a matched-pair control group. Patients undergoing this procedure are likely able to return at the same level of sport before surgery.

## 1. Introduction

Chondral lesions in hip joints are a common cause of morbidity in young patients, especially in athletes, that may lead to early degenerative articular changes and osteoarthritis and eventually requiring total hip replacement [1]. In particular, the acetabulum is prone to chondral lesions secondary to trauma, hip dysplasia, and femoral acetabular impingement (FAI). Despite the high frequency of these lesions, still no consensus on the correct management has been reached. Indeed, for focal and full-thickness acetabular lesions, three main approaches have been proposed: tissue repair, tissue transplant, and creation of a biologically favorable environment for cartilage repair [2]. Arthroscopic microfractures fall in the latter group and address cartilage defects by drilling the underlying bone in order to allow bone marrow, progenitor cells and growth factors, to fill the defect and promote tissue regeneration [3,4]. This technique, thanks to advances in the field of hip arthroscopy, has become easier to perform and more reproducible as described in the literature [5]. A number of studies investigated the efficacy of microfractures on acetabular cartilage lesions comparing it to other approaches, and all of them showed its non-inferiority in terms of patient reported outcomes (PRO) [6,7,8,9]. Nevertheless, since this procedure is often performed in young athletic patients, the return to sport activity rate represents a crucial outcome to be investigated. Indeed, only one study attempted to explore this aspect, finding a higher rate of return to sport in the non-microfracture group (84%) compared to the microfracture group (77%), but the reliability of the results is limited by the small number of included patients (only 39) [10]. Therefore, the aim of the present study was to investigate the return to sport activity and clinical outcomes at two years after surgery in patients undergoing arthroscopic acetabular microfracture for a grade IV chondral lesion compared to patients without full-thickness chondral damage.

## 2. Materials and Methods

This retrospective matched-pair controlled study was approved by the Ethical Committee Board of our Institution (Humanitas Research Hospital). We included in the study group all patients undergoing hip arthroscopy with acetabular microfracture for a full-thickness chondral lesion (Outerbridge grade IV) in the context of none or mild osteoarthritis (Tönnis grade 0–1). The control group included all patients that underwent hip arthroscopy without microfracture for a Grade III or less chondral damage of the acetabulum. All included patients were operated on in our tertiary referral center between January 2011 and March 2016. The study group and the control group were paired on a 1:1 ratio based on age and gender. The comparison of the baseline features of the two groups is presented in Table 1. Exclusion criteria included previous hip arthroscopy of the ipsilateral hip, Tönnis grade > 2 and follow-up less than two years. Reasons for hip arthroscopy were the management of labral tear, FAI, chondral pathology, ligamentum teres injuries, capsule pathology, and/or removal of loose bodies. Clinical evaluation and outcomes assessment were performed by two non-blinded independent authors (X.X. and X.X. pre- and postoperatively at the last available follow-up with patient-reported outcome (PRO) scores such as modified Harris Hip Score (mHHS) and the Hip Disability and Osteoarthritis Outcome Score (HOOS) [11,12]. According to mHHS, the results were excellent with a score of 90–100, good with 80–89, fair with 70–79, poor with 60–69, failed below 60. Pain was estimated on a visual analogue scale (VAS) from 0 to 10 with higher scores indicating the worst conditions. For each patient, the participation in sports was evaluated based on a questionnaire assessing the type and level of activities preoperatively and at a minimum two years of follow-up. The level of impact of sports was scored as low, potentially low, intermediate or high, adapting the classification of Clifford and Mallon [13]. The level of sport activity was defined as either amateur or professional based on the available patients’ medical records. In particular, a patient was defined “amateur” if he performed on average ≤ 2 training per week, and “elite” if he either performed > 2 training per week or played at professional level. All patients underwent preoperative plain radiographs including an anteroposterior pelvic view, Dunn view, cross-table lateral view, and a false profile view [14]. A preoperative magnetic resonance of the hip was also available for all patients. The imaging studies were retrospectively reviewed by two blinded orthopedic surgeons (X.X. and X.X.). The cam-type FAI was defined by an alpha angle greater than 60° [1]. The pincer-type FAI was defined by a lateral center-edge angle greater than 40° [15], a positive crossover sign, coxa profunda, or protrusio acetabuli. Grading of the chondral damage was performed according to the Outerbridge grading scale18, while the classification of osteoarthritis was evaluated with the Tönnis grading system [16]. The articular rim height and the size of the defect were measured intraoperatively, and the latter was classified as follows: small (<200 mm^2^), medium (≥200 and <350 mm^2^), and large (≥350 mm^2^).

### 2.1. Surgical Procedure

Written informed consent was obtained from each patient before the surgical procedure. All hip arthroscopies were performed by one experienced surgeon with the patient in the supine position and using a minimum of 3 portals (anterior, midanterior, and anterolateral) for the assessment of both the central and peripheral compartments of hip joints. All patients received prophylactic antibiotics and routine postoperative thromboembolic prophylaxis with low molecular weight heparin. Pincer-type and cam-type impingement were managed with acetabuloplasty and femoral neck osteoplasty, respectively, under fluoroscopic guidance. According to the type of lesion, labral tears were repaired or selectively debrided preserving as much stable labrum as possible. The location of the chondral defect was reported by using a clock-face description where 12 o’clock indicated the side towards the head of patient, and 6 o’clock was the side towards the obturator foramen [2,4]. The length and the width of the chondral defects were measured using an arthroscopic probe with 5 mm markings. In the microfracture group, the cartilage lesion was managed by removing with an arthroscopic shaver the loose articular flaps and delaminated cartilage covering the defect. Subsequently, the subchondral bony surface was exposed, and perpendicular and stable borders of the lesion were created with a ring curette. The microfractures were performed perpendicular to the subchondral bone with an angled, calibrated arthroscopic awl. Multiple holes were made 3 to 4 mm apart to a depth of 3 to 4 mm in the exposed subchondral bone plate [1,2]. The back bleeding from the holes, under interruption of the fluid inflow, was considered as evidence of the appropriate penetration of the awl’s tip in the subchondral bone plate.

### 2.2. Postoperative Rehabilitation

The patients were allowed to stand on the first postoperative day, and to walk with crutches and no weight-bearing (microfracture group) or partial weight-bearing (control group) on the operated lower limb for the first four weeks. The physical therapy was started on the first postoperative day with range of motion exercises. Moreover, the use of a continuous passive motion machine for 4 h per day was recommended in all operated patients. The rehabilitation program with range of motion and abductor exercises was performed for four weeks. After this period, the patients started gradually increasing the weight-bearing on the operated lower limb to full weight-bearing.

### 2.3. Statistical Analysis

Continuous variables were expressed as mean and standard deviation (SD) or range. Categorical variables were expressed as proportion and percentage. Before performing the statistical analysis, a sample size calculation was performed to estimate the number of cases needed. A difference of 10 points in the mHHS was considered significant based on a previous study 11. Post hoc analysis of the power result using 62 patients with effect size 0.5, is 0.8. Therefore, with an estimated SD of 6, the effect size for the 2-tailed Student *t* test in order to obtain a power of the study at 80% and an α-error of 5%, the clinical study needed a minimum of 42 cases in each group to achieve statistical significance. The normal distribution of the data was confirmed with the Kolmogorov–Smirnov test. The 2-tailed Student’s *t* test was used to compare the preoperative and last follow-up values of mHHS and HOOS. A regression analysis was used to compare last follow-up values of mHHS and HOOS between subgroups of patients according to age, body mass index (BMI), articular rim height, postoperative sport level, postoperative type of sport, and postoperative sport impact. The one-way ANOVA was performed to compare the last follow-up values of mHHS and HOOS between and within groups according to size of the defect and grade of Tönnis. The multiple comparisons between the groups were performed with the Tukey post hoc test. All the analyses were performed using SPSS for Mac (version 21.0, SPSS Inc., Chicago, IL, USA). A *p* value of <0.05 was considered significant.

## 3. Results

### 3.1. Demographics

In a database of 630 consecutive hip arthroscopies from 2011 to 2017, 62 patients (M:F = 58:4) (62 hips) who had acetabular microfracture were included and matched with 62 patients (62 hips) who underwent hip arthroscopy alone. In the microfracture group, the preoperative diagnosis was: cam-type FAI in 43 (69.4%) patients and combined FAI in 16 (25.8%). In the control group, the preoperative diagnosis was: cam-type FAI in 41 (66.1%) patients, and combined FAI in 21 (33.9%). The statistical analysis comparing the prevalence of cam-type FAI (*p* = 0.70) and combined FAI (*p* = 0.33) in the two groups resulted in no statistically significant differences, implying a baseline similar condition in the study and control groups. At the time of surgery, the average age was 35.1 ± 8.1 years (range, 17–52) in the microfracture group and 36.4 ± 6.3 (range, 21–64) in the control group and the difference was not significant. The average BMI was 25.6 ± 2.5 kg/cm^2^ (range, 20–32) in the microfracture group and 25.9 ± 3.2 (range, 21–31) in the control group (n.s.). The average follow-up was 53.74 ± 18.3 months (range, 24–86) in the microfracture group and 52.8 ± 12.1 months (range, 24–84) in the control group (n.s.). The average duration of symptoms before surgery was 26.3 ± 20.2 months (range, 2–84) in the microfracture group and 25.3 ± 20.2 months (range, 2–84) in the control group (n.s.). The average articular rim height was 3.2 ± 0.9 mm (range, 1.7–5.9) in the microfracture group and 3.4 ± 1.2 mm (1.9–6.1) in the control group (n.s.). In Table 1 are reported the arthroscopic procedures performed in the two groups, the frequency of these was similar in the two groups save for a statistically significant higher rate in the control group of radiofrequency labral stabilization, femoral osteoplasty, and transcapsular iliopsoas tenotomy. The average size of the lesions was 132 mm^2^ (range, 43–620).

### 3.2. Sport Activity

In the microfracture group, the number of patients playing at the amateur and elite level preoperatively was 38 (66.6%) and 19 (33.4%), respectively, and 49 (74.5%) and 9 (25.5%) at the last follow-up. One patient did not participate in sport before surgery but performed sport at amateur level at the last follow-up. Instead, in the control group, the number of patients playing at the amateur and elite level preoperatively was 37 (64.9%) and 20 (35.1%), respectively, and 45 (78.9%) and 12 (21.1%) at the last follow-up. The change in number of elite participants in sport activity in the two groups at last available follow up was not significant (*p* = 0.66). The sports participation according to Clifford, is reported in Table 2 taking into account type and level of impact of activities preoperatively and at the last follow-up. Notably, preoperatively, 74% of patients in the microfracture group and 77% of patients in the control group practiced sports at high or intermediate impact; while postoperatively it dropped to 48% in the microfracture group and to 49% in the control group, the difference was not statistically significant (*p* = 0.75).

### 3.3. Clinical Scores

In the microfracture group, the average mHHS changed from 71.3 ± 6.5 preoperatively to 89.3 ± 5.1 at the last follow-up (<0.001), whereas the average HOOS changed from 78.2 ± 6.4 to 87.9 ± 6.7 (<0.001), and the average VAS changed from 5.4 ± 2.6 to 3.1 ± 2.2 (<0.001). Instead, in the control group, the average mHHS changed from 69.7 ± 8.2 preoperatively to 87.9 ± 8.3 at the last follow-up (<0.001), whereas the average HOOS changed from 79.4 ± 7.8 to 87.6 ± 10.5 (<0.001), and the average VAS changed from 5.2 ± 3.7 to 3.4 ± 2.8 (<0.001). At last follow-up, no differences were reported between the two groups in terms of average value for mHHS (*p* = 0.26), HOOS (*p* = 0.85), and VAS (*p* = 0.5).

In the microfracture group, age, BMI, Tönnis’ grade and articular rim height did not affect the last follow-up mHHS and HOOS (Table 3). On the other hand, the size of the defect affected the last follow-up HOOS with higher scores in patients with smaller lesions (Table 3). Finally, there was no significant difference in terms of mHHS and HOOS according to postoperative sport activity (Table 4).

### 3.4. Complications

In both groups, there were no complications, including infection, thromboembolism, paresthesia, or peripheral neurologic deficits. In the microfracture group, one (1.6%) patient underwent revision arthroscopy, and two (3.2%) patients underwent total hip replacement; in the control group, one (1.6%) patient underwent revision arthroscopy, and one (1.6%) patient underwent total hip replacement.

## 4. Discussion

The main finding of the present study was that acetabular microfracture for Outerbridge grade IV lesions provided significant improvement in clinical outcomes in terms of PRO scores at a minimum follow-up of two years. Moreover, the operated patients were able to return to the same level of sport before surgery, with only 17% of them participating in a lower level. When compared to the control group, these results were not statistically different both in terms of PRO score and in sport participation change. In addition, we observed that preoperative features of patients such as age, BMI, articular rim height, size of the defect, and grade of Tönnis, and postoperative sport activity did not affect the PRO scores in patients undergoing microfractures of the acetabulum. In the present study, we included all patients with Outerbridge grade IV full-thickness defect with 92% of them having small/medium size lesions (up to 350 mm^2^). According to the PRO scores, we found excellent/good results according to the mHHS score in 95% of patients. Some authors previously investigated the results of hip arthroscopy with microfracture to manage chondral defects with similar average size, reporting excellent/good results in 61% to 85% of patients [7,9,10,14,17]. This difference may be explained in light of the variability of the included patients in each study. Indeed, the included patients of the current work were involved in both elite and amateur sport activity, while in J. E. McDonalds et al.’s study15 they were all participating in elite sport activity. Therefore, the former may have had lower functional requests compared to the elite-only cohort of patients. Moreover, the cohorts investigated by Karthikeyan S. et al. [7] and Trask D.J. et al. [9] had, respectively, a larger mean acetabular chondral defect (154 mm^2^ and 143 mm^2^) compared to the study group described in this work (132 mm^2^). Indeed, a negative correlation between the chondral defect size and the PRO has been observed in the current study and can be visualized in Table 3. In our study, we also reported revision arthroscopy and conversion in total hip replacement (THR) in 1.6% and 3.2%, respectively. On the other hand, in previous studies [7,9,10,14,17], the rate of revision arthroscopy ranged from 7% to 34% and the rate of conversion in THR ranged from 0% to 17%. This difference in rate of conversion to revision arthroscopy or THR between the current study and the available literature may be explained once again by the difference in chondral defect size as described above. Moreover, the baseline preoperative osteoarthritis level possibly played a role in determining the timing of revision surgery. In particular, in the current study, an inclusion criterion was Tönnis score ≤ 1, while in Karthikeyan S. et al. [7], it the baseline degree of osteoarthritis was not reported. Trask and Keene19 reported that the clinical results of the microfracture procedure are not affected by age and size of defect. In our study, we found slightly higher PRO scores in the group of small-size defects compared with groups of medium- and large-size defects. However, this difference did not appear clinically relevant. Finally, we also demonstrated that postoperative PRO scores are not affected by preoperative Tönnis’ grade and articular rim height. McDonald et al. [10] investigated 39 elite male athletes undergoing hip arthroscopy with microfracture for Outerbridge grade IV chondral lesions. They reported a return to play in 77% of patients with no statistically significant difference when compared to a control group of elite athletes managed without microfracture. In our study, patients were able to maintain the same level of sport before surgery in 83% of cases. The higher rate in return to the same level of sport observed in our cohort may be explained by the fact that we included both elite and amateur athletes while McDonald et al. only included elite patients, for which returning to the same level of sport activity is a harder challenge compared to amateur athletes. Indeed, if we extract the data specifically for amateur athletes, the rate of return to same level of sport was 100%, while for elite athletes this percentage dropped to 36%. The results of this study suggest also that hip arthroscopy with microfracture could be indicated also in patients with an age >40 years and lesions with a size >350 mm^2^. Although non-contact and light impact sports may be less demanding on the hip joint, good clinical outcomes can also be expected in patients participating in contact and high impact sports after surgery. Finally, the risk of conversion in total hip replacement is relatively low regardless of the postoperative sport activity. A major strength of the study is represented by matched-pair control design including patients with acetabular chondral defect operated by the same surgeon and prospectively evaluated with PRO scores in a period of at least two years. In this respect, no patients were lost to follow-up. Another strength consisted in taking into account potential confounders for the results of surgery, such as age, BMI, articular rim height, size of the defect, grade of Tönnis, and postoperative sport activity. To the best of our knowledge, this is the first study to evaluate the effect of BMI, preoperative arthritis as demonstrated by articular rim height and Tönnis’ grade, and postoperative sport activity on the clinical outcomes after microfracture. We are aware that the present study has some limitations. First of all, it is a retrospective cohort study, and all clinical data were retrospectively collected from medical records of hospital stay and follow-up consultations. Secondly, the presence of other arthroscopic procedures performed in our patients during the index surgery prevented us from evaluating the effect specifically due to microfracture. Moreover, the control group had a grade III or less chondral damage of the acetabulum according to the Outerbridge classification, while the microfracture group had a grade IV. However, considered the efficacy of microfractures in our clinical practice, we treated all patients with Grade IV.

## 5. Conclusions

In conclusion, hip arthroscopy with microfractures for the management of full-thickness acetabular chondral defect provides good clinical results at a minimum follow-up of two years. PRO scores are not inferior with respect to a matched-pair control group. Patients undergoing this procedure are likely able to return at the same level of sport before surgery.

## Figures and Tables

**Table 1:** Procedures performed during hip arthroscopy in both microfracture and control groups. The results of the statistical analysis comparing the frequency of these procedures in the two groups are reported in the last column as *p* values.

Surgical Procedure	Microfracture Group	Control Group	*p*-Value
Labral debridement	1 (1.6%)	-	0.274
Labral repair	35 (56.5)	31 (51.6%)	0.471
Labral repair with fascia lata	1 (1.6%)	2 (3.2%)	0.558
Partial anterior limbectomy	1 (1.6%)	3 (4.8%)	0.309
Ligamentum teres debridement	1 (1.6%)	-	0.274
Radiofrequency labral stabilization	2 (3.2%)	4 (6.5%)	0.402
Radiofrequency cartilage stabilization	-	6 (9.7%)	**0.012**
Removal of os acetabuli	1 (1.6%)	1 (1.6%)	1
Posterior capsular release	1 (1.6%)	-	0.274
Transcapsular iliopsoas tenotomy	-	5 (8.1%)	**0.022**
Loose body removal	-	1 (1.6%)	0.274
Synovectomy	-	2 (3.2%)	0.153
Femoral osteoplasty	61 (98.4%)	54 (87.1%)	**0.015**
Acetabular osteoplasty	13 (20.9%)	12 (19.4%)	0.822

**Table 2:** Sport participation preoperatively and at the last follow-up in both microfracture and control groups, subdivided according to the level of impact according to the Clifford classification.

Type	Microfracture Group		Control Group	
	N Preoperative	N Postoperative	N Preoperative	N Postoperative
***High***				
Soccer	14	2	22	4
Basketball	3	3	1	0
Kickboxing	1	1	1	1
Martial arts	1	0	3	0
Volleyball	1	1	4	2
Triathlon	3	2	1	1
Skiing	1	1	3	4
Running	16	9	7	9
Motocross	0	0	1	0
***Intermediate***				
Tennis	2	1	1	1
***Potentially low***				
Cycling	3	8	3	9
Gym	5	10	5	16
***Low***				
Swimming	4	17	3	13
Dancing	1	1	2	4
Spinning	1	1	0	0
Golf	1	1	0	0

**Table 3:** Clinical outcomes at last follow-up according to preoperative features of patients and chondral defects. Legend: mHHS: modified Harris Hip Score; HOOS: Hip Disability and Osteoarthritis Outcome Score; FU: follow-up; BMI: Body Mass Index.

Variables	N of Patients (%)	Last FU mHHS (Mean, SD)	*p* Value	Last FU HOOS (Mean, SD)	*p* Value
Age					
≥40	20 (32.3%)	89.8 ± 3.5	0.53	87.1 ± 5.9	0.47
<40	42 (67.7%)	89.1 ± 5.8		88.3 ± 6.9	
BMI					
≥25	39 (62.9%)	89.5 ± 5.0	0.47	87.3 ± 7.1	0.38
<25	22 (37.1%)	88.5 ± 5.0		88.2 ± 5.9	
Defect size					
small	41 (66.1%)	88.7 ± 5.1	0.15	92.0 ± 1.4	**0.048**
medium	16 (25.8%)	91.3 ± 4.8		84.7 ± 10.5	
large	5 (8.1%)	88.0 ± 5.4		88.6 ± 4.4	
Tönnis					
0	25 (38.7%)	88.8 ± 5.8	0.85	87.8 ± 5.7	0.83
1	33 (53.2%)	89.6 ± 4.5		89.7 ± 7.1	
2	4 (6.5%)	89.7 ± 8.1		89.6 ± 1.7	
Articular rim height					
≥2 mm	52 (83.8%)	89.1 ± 5.4	0.39	87.7 ± 7.1	0.65
<2 mm	10 (15.2%)	90.4 ± 3.9		88.4 ± 3.7	

**Table 4:** Clinical outcomes at last follow-up according to postoperative sport activity. Legend: mHHS: modified Harris Hip Score; HOOS: Hip Disability and Osteoarthritis Outcome Score; FU: follow-up; BMI: Body Mass Index.

Variables	N of Patients (%)	Last FU mHHS (Mean, SD)	*p* Value	Last FU HOOS (Mean, SD)	*p* Value
Postoperative sport level					
elite	9 (25.5%)	87.9 ± 6.1	0.58	87.9 ± 13.1	0.98
amateur	49 (74.5%)	89.1 ± 4.8		87.8 ± 5.1	
Postoperative contact sport					
yes	6 (9.3%)	88.2 ± 4.9	0.71	90.5 ± 3.9	0.15
no	52 (90.7%)	89.0 ± 5.0		87.5 ± 7.0	
Postoperative sport impact					
high	20 (34.5%)	89.9 ± 4.2	0.27	89.7 ± 7.7	0.08
light	38 (65.5%)	88.4 ± 5.3		86.8 ± 4.4	

## References

[1] Agricola R., Waarsing J.H., Thomas G.E., Carr A.J., Reijman M., Bierma-Zeinstra S.M.A., Glyn-Jones S., Weinans H., Arden N.K. (2014). Cam Impingement: Defining the Presence of a Cam Deformity by the Alpha Angle: Data from the CHECK Cohort and Chingford Cohort. Osteoarthr. Cartil..

[2] Bagheri K., Sierra F., Jamali A.A. (2020). Acetabular Cartilage Repair: State of the Art in Surgical Treatment. J. Hip. Preserv. Surg..

[3] Haughom B.D., Erickson B.J., Rybalko D., Hellman M., Nho S.J. (2014). Arthroscopic Acetabular Microfracture with the Use of Flexible Drills: A Technique Guide. Arthrosc. Tech..

[4] Green C.J., Beck A., Wood D., Zheng M.H. (2016). The Biology and Clinical Evidence of Microfracture in Hip Preservation Surgery. J. Hip. Preserv. Surg..

[5] Maldonado D.R., Chen J.W., Lall A.C., Kyin C., Walker-Santiago R., Shapira J., Rosinsky P.J., Domb B.G. (2019). Microfracture in Hip Arthroscopy. Keep It Simple!. Arthrosc. Tech..

[6] Hevesi M., Bernard C., Hartigan D.E., Levy B.A., Domb B.G., Krych A.J. (2019). Is Microfracture Necessary? Acetabular Chondrolabral Debridement/Abrasion Demonstrates Similar Outcomes and Survival to Microfracture in Hip Arthroscopy: A Multicenter Analysis. Am. J. Sports Med..

[7] Karthikeyan S., Roberts S., Griffin D. (2012). Microfracture for Acetabular Chondral Defects in Patients with Femoroacetabular Impingement: Results at Second-Look Arthroscopic Surgery. Am. J. Sports Med..

[8] Kester B.S., Begly J.P., Capogna B., Chenard K., Youm T. (2020). Four-Year Outcomes Following Arthroscopic Microfracture of the Hip in Patients with Advanced Chondral Lesions. Bull. Hosp. Jt. Dis..

[9] Trask D.J., Keene J.S. (2016). Analysis of the Current Indications for Microfracture of Chondral Lesions in the Hip Joint. Am. J. Sports Med..

[10] McDonald J.E., Herzog M.M., Philippon M.J. (2013). Return to Play after Hip Arthroscopy with Microfracture in Elite Athletes. Arthroscopy.

[11] Hinman R.S., Dobson F., Takla A., O’Donnell J., Bennell K.L. (2014). Which Is the Most Useful Patient-Reported Outcome in Femoroacetabular Impingement? Test-Retest Reliability of Six Questionnaires. Br. J. Sports Med..

[12] Martin R.L., Philippon M.J. (2007). Evidence of Validity for the Hip Outcome Score in Hip Arthroscopy. Arthroscopy.

[13] Clifford P.E., Mallon W.J. (2005). Sports after Total Joint Replacement. Clin. Sports Med..

[14] Domb B.G., Gupta A., Dunne K.F., Gui C., Chandrasekaran S., Lodhia P. (2015). Microfracture in the Hip: Results of a Matched-Cohort Controlled Study With 2-Year Follow-Up. Am. J. Sports Med..

[15] Sankar W.N., Matheney T.H., Zaltz I. (2013). Femoroacetabular Impingement: Current Concepts and Controversies. Orthop. Clin. N. Am..

[16] Kovalenko B., Bremjit P., Fernando N. (2018). Classifications in Brief: Tönnis Classification of Hip Osteoarthritis. Clin. Orthop. Relat. Res..

[17] Byrd J.W.T., Jones K.S. (2009). Arthroscopic Femoroplasty in the Management of Cam-Type Femoroacetabular Impingement. Clin. Orthop. Relat. Res..

